# Supplementary material for: Enhanced virulence and stress tolerance are signatures of epidemiologically successful Shigella sonnei
Source: Nat Commun. 2025 Oct 9;16:9005. doi: 10.1038/s41467-025-64057-y (PMC12511311; doi:10.1038/s41467-025-64057-y)
Supplement: Supplementary file 1 — Supplementary Information [file 41467_2025_64057_MOESM1_ESM.pdf]

Supplemental information

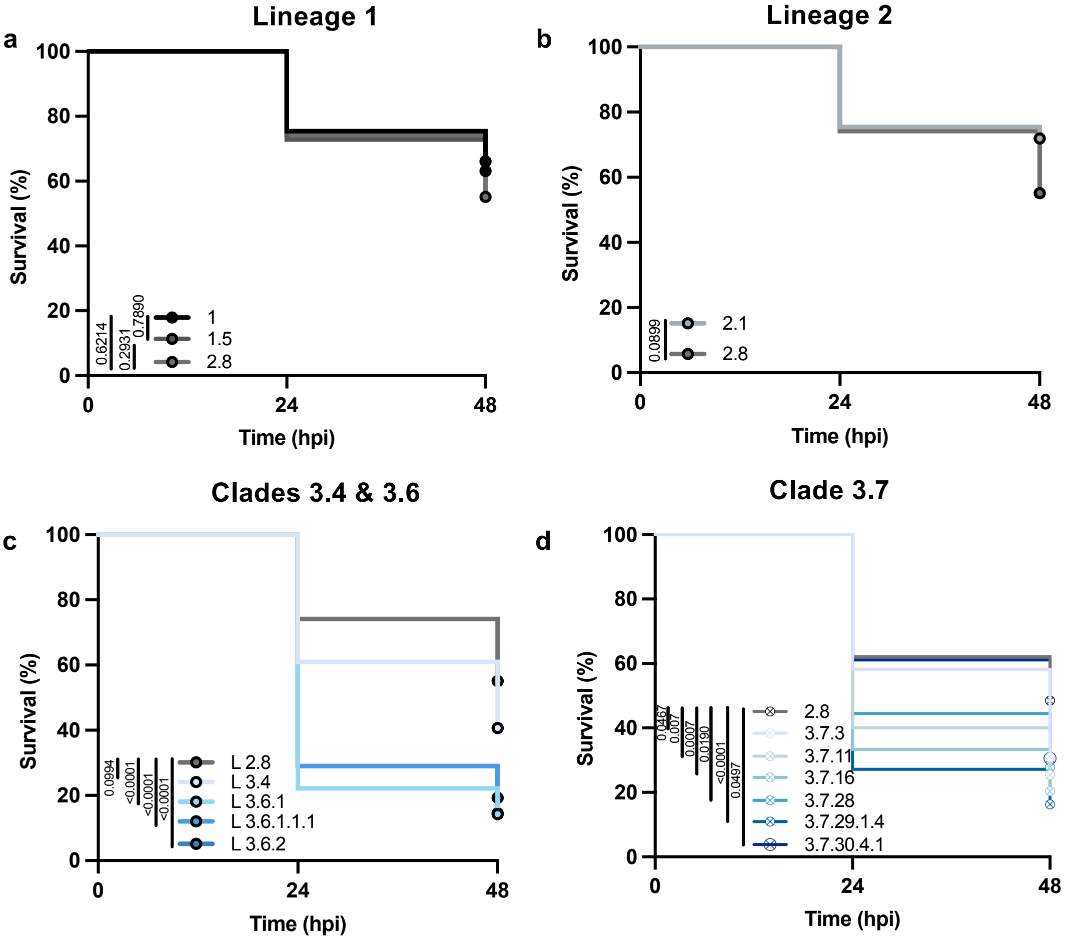

Figure S1. Inclusion of additional representatives from each lineage reinforce that Lineage 3 is more virulent in zebrafish. Survival of zebrafish larvae infected with Lineage 1 isolates **(a)**, Lineage 2 isolates **(b)**, Clades 3.4 and 3.6 **(c)** and Clade 3.7 **(d)**. Lineage 2.8 was included in all experiments for comparative purposes. N=3 biological replicates, with ≥12 larvae per condition/experiment. **Statistics:** log-rank (Mantel-Cox) test. Source data are provided as a Source Data file.

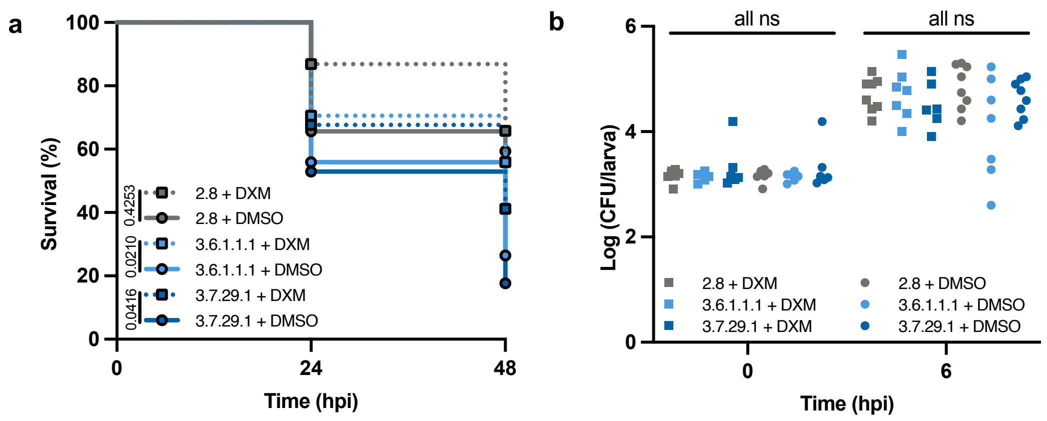

Figure S2. Chemical suppression of inflammation partly rescues the survival of *S. sonnei* infected zebrafish larvae. Zebrafish larvae were infected and bathed in either 50 µg/mL dexamethasone (DXM) (a chemical suppressant of inflammation), or an equal concentration of DMSO as a negative control. **(a)** Survival of infected zebrafish bathed in DXM or DMSO, survival was measured at 24 and 48 hpi. N=2 biological replicates, with ≥12 larvae per condition/experiment (mean ± SEM). **(b)** Colony forming units (CFU) were enumerated at 0 and 6 hpi by the mechanical disruption of larvae, and plating of homogenate, N=2 biological replicates with ≥ 4 larvae per group (mean ± SEM). **Statistics:** log-rank (Mantel-Cox) test (a), two-way ANOVA with Tukey’s correction applied (b) ns = not significant. Source data are provided as a Source Data file.

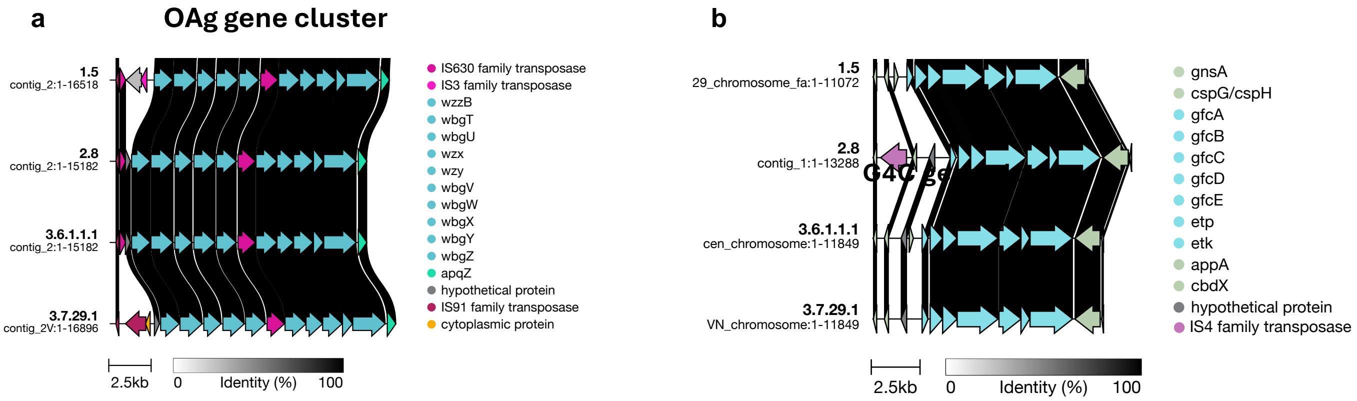

Figure S3. Gene cluster alignment reveals variations in upstream insertion sequence content. Gene clusters were aligned and visualised using Clinker. **(a)** Alignment of pINV encoded O-antigen encoding genomic clusters. **(b)** Alignment of chromosomally encoded group four capsule (G4C) encoding genomic clusters.

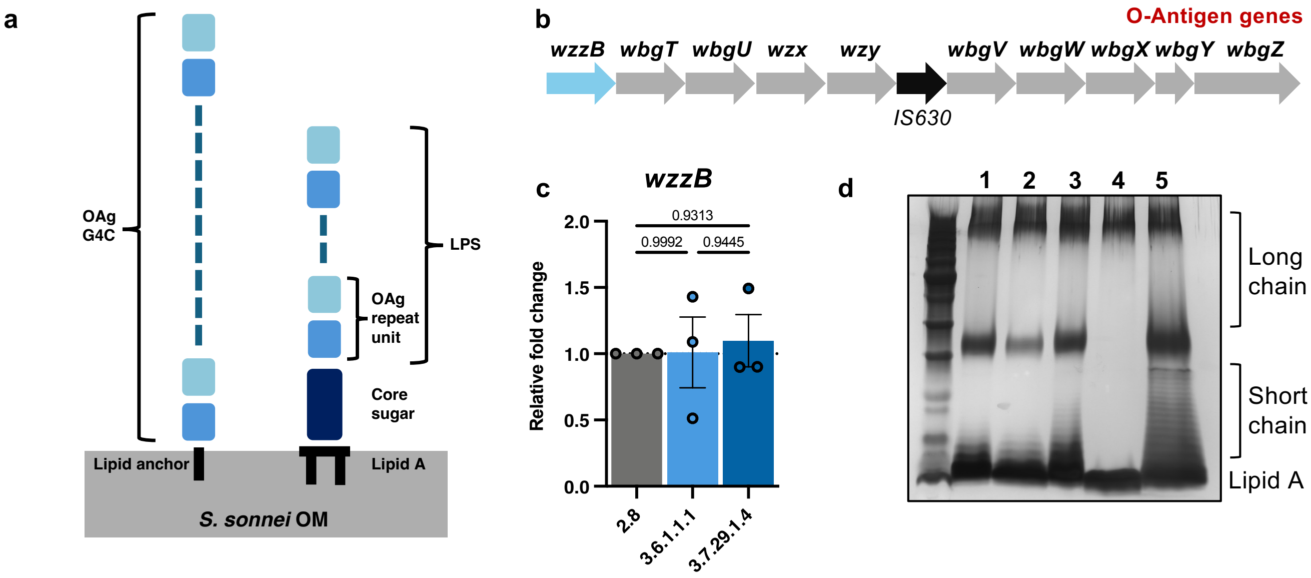

Figure S4. Lipopolysaccharide expression or composition does not differ in Lineage 3 *S. sonnei*. (a) Schematic of outer membrane surface polysaccharides present in *S. sonnei*. **(b)** Schematic of pINV encoded O-antigen synthesis gene cluster. **(c)** Relative mRNA expressions of *wzzB* analysed through qRT-PCR, N=3, technical duplicates (mean ± SEM). **(d)** Visualisation of LPS extracts using SDS-PAGE and silver staining. Lane 1 = 2.8, 2= 3.6.1.1.1, 3 = 3.7.29.1.4, 4 = Δ*waal* (O-antigen mutant), 5 = ΔG4C (group four capsule mutant). **Statistics:** one-way ANOVA, Tukey's correction applied. Source data are provided as a Source Data file.

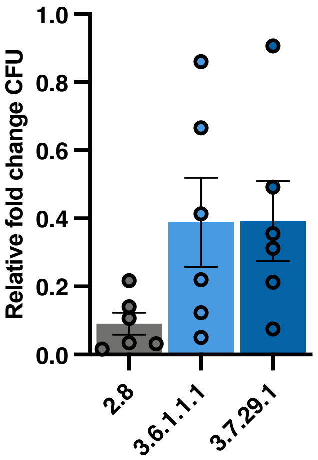

Figure S5. Relative bacterial burden from infected neutrophils. Colony forming units (CFU) obtained from infected neutrophils at 1 hour post infection (hpi) relative to the inoculum dose. A value less than 1 represents a decrease in the total bacterial burden following infection, compared to the inoculum dose. N=6 biological replicates (mean ± SEM). Source data are provided as a Source Data file.
