# Supplementary material for: Enhanced virulence and stress tolerance are signatures of epidemiologically successful Shigella sonnei
Source: Nat Commun. 2025 Oct 9;16:9005. doi: 10.1038/s41467-025-64057-y (PMC12511311; doi:10.1038/s41467-025-64057-y)
Supplement: Supplementary file 2 — Reporting Summary [file 41467_2025_64057_MOESM2_ESM.pdf]

nature portfolio

Corresponding author(s): Prof. Serge Mostowy

Last updated by author(s): 25/08/25

Reporting Summary

Nature Portfolio wishes to improve the reproducibility of the work that we publish. This form provides structure for consistency and transparency in reporting. For further information on Nature Portfolio policies, see our Editorial Policies and the Editorial Policy Checklist.

Statistics

For all statistical analyses, confirm that the following items are present in the figure legend, table legend, main text, or Methods section.

n/a	Confirmed	
☐	☒	The exact sample size (n) for each experimental group/condition, given as a discrete number and unit of measurement
☐	☒	A statement on whether measurements were taken from distinct samples or whether the same sample was measured repeatedly
☐	☒	The statistical test(s) used AND whether they are one- or two-sided<br>*Only common tests should be described solely by name; describe more complex techniques in the Methods section.*
☒	☐	A description of all covariates tested
☒	☐	A description of any assumptions or corrections, such as tests of normality and adjustment for multiple comparisons
☐	☒	A full description of the statistical parameters including central tendency (e.g. means) or other basic estimates (e.g. regression coefficient) AND variation (e.g. standard deviation) or associated estimates of uncertainty (e.g. confidence intervals)
☐	☒	For null hypothesis testing, the test statistic (e.g. F, t, r) with confidence intervals, effect sizes, degrees of freedom and P value noted<br>*Give P values as exact values whenever suitable.*
☒	☐	For Bayesian analysis, information on the choice of priors and Markov chain Monte Carlo settings
☒	☐	For hierarchical and complex designs, identification of the appropriate level for tests and full reporting of outcomes
☒	☐	Estimates of effect sizes (e.g. Cohen's d, Pearson's r), indicating how they were calculated

Our web collection on statistics for biologists contains articles on many of the points above.

Software and code

Policy information about availability of computer code

Data collection	Fluorescence microscopy was performed using a ZEISS CellDiscover 7 (CD7) microscope using a ZEISS Plan-APOCHROMAT 20× / 0.95 Autocorr Objective coupled to a 0.5x tubelens, driven by ZEN blue software (v3.5)
Data analysis	ABRicate (v.1.0.1)<br>MAFFT (v.7.526)<br>FastTree (v.2.1.1)<br>Phandango (accessed on 19/12/2024)<br>Inkscape (v.1.3.2)<br>Clinker (v.0.0.29)<br>Graphpad Prism (v.10.5.0)<br>Mauve (v.2.4.0)

For manuscripts utilizing custom algorithms or software that are central to the research but not yet described in published literature, software must be made available to editors and reviewers. We strongly encourage code deposition in a community repository (e.g. GitHub). See the Nature Portfolio guidelines for submitting code & software for further information.

Data

Policy information about availability of data

All manuscripts must include a data availability statement. This statement should provide the following information, where applicable:

- Accession codes, unique identifiers, or web links for publicly available datasets
- A description of any restrictions on data availability
- For clinical datasets or third party data, please ensure that the statement adheres to our policy

All data are included in the source data associated with this manuscript. Biological materials can be obtained from the corresponding author.

Research involving human participants, their data, or biological material

Policy information about studies with human participants or human data. See also policy information about sex, gender (identity/presentation), and sexual orientation and race, ethnicity and racism.

Reporting on sex and gender	This information was not collected.
Reporting on race, ethnicity, or other socially relevant groupings	See above.
Population characteristics	See above.
Recruitment	See above.
Ethics oversight	Human neutrophil experiments were conducted under research ethics committee reference 17/LO/1531.

Note that full information on the approval of the study protocol must also be provided in the manuscript.

Field-specific reporting

Please select the one below that is the best fit for your research. If you are not sure, read the appropriate sections before making your selection.

☒ Life sciences ☐ Behavioural & social sciences ☐ Ecological, evolutionary & environmental sciences

For a reference copy of the document with all sections, see nature.com/documents/nr-reporting-summary-flat.pdf

Life sciences study design

All studies must disclose on these points even when the disclosure is negative.

Sample size	No statistical methods were used to predetermine sample size. Sample sizes were chosen based on previous experience with each assay.
Data exclusions	No data were excluded from the analysis.
Replication	All experiments were performed at least three independent times (except for Figures S2A and S2B), there were no issues with replication.
Randomization	Cells were taken from at liquid cultures at random. Zebrafish embryos were randomly selected for infection.
Blinding	Investigators were not blinded during data collection, all processes were performed by the same person.

Reporting for specific materials, systems and methods

We require information from authors about some types of materials, experimental systems and methods used in many studies. Here, indicate whether each material, system or method listed is relevant to your study. If you are not sure if a list item applies to your research, read the appropriate section before selecting a response.

Materials & experimental systems

n/a	Involved in the study	
☐	☒	Antibodies
☐	☒	Eukaryotic cell lines
☒	☐	Palaeontology and archaeology
☐	☒	Animals and other organisms
☒	☐	Clinical data
☒	☐	Dual use research of concern
☒	☐	Plants

Methods

n/a	Involved in the study	
☒	☐	ChIP-seq
☒	☐	Flow cytometry
☒	☐	MRI-based neuroimaging

Antibodies

Antibodies used	AlexaFluor-647 conjugated Phalloidin (1:500, #10656353, ThermoFisher Scientific)
Validation	All antibodies used in this study have been used in previous publications (Mostowy et al, Cell Host Microbe, 2010; Krokowski et al, Cell Host Microbe, 2018; Lobato-Marquez et al, Nature Communciations, 2021)

Eukaryotic cell lines

Policy information about cell lines and Sex and Gender in Research

Cell line source(s)	HeLa cells were obtained from ATCC.
Authentication	HeLa cells were obtained from ATCC. Cell lines were not authenticated.
Mycoplasma contamination	Cell lines were tested for Mycoplasma infection and tested negative.
Commonly misidentified lines (See ICLAC register)	No commonly misidentified cell lines were used.

Animals and other research organisms

Policy information about studies involving animals; ARRIVE guidelines recommended for reporting animal research, and Sex and Gender in Research

Laboratory animals	The study involved the use zebrafish (Danio rerio) embryos under 5 days post fertilisation which are not regulated under UK law.
Wild animals	This study did not involve wild animals.
Reporting on sex	This data was not collected as only embryos were used.
Field-collected samples	This study did not involve field-collected samples.
Ethics oversight	Animal experiments were performed according to the Animals (Scientific Procedures) Act 1986 and approved by the Home Office (Project licenses: PPL P84A89400 and P4E664E3C).

Note that full information on the approval of the study protocol must also be provided in the manuscript.

Plants

Seed stocks	N/A
Novel plant genotypes	N/A
Authentication	N/A
